# Epitranscriptomic regulation by m6A in immunity and autoimmune disorders: emerging mechanisms and clinical perspectives

**DOI:** 10.3389/fimmu.2026.1887190

**Published:** 2026-07-03

**Authors:** Madiha Maqsood, Chunai Zhan, Muhammad Hassan, Xinyu Li, Long Mei, Boyang Yang, Wenwen Zhu, Wei Shao

**Affiliations:** 1 Department of Microbiology and Parasitology, Anhui Provincial Laboratory of Pathogen Biology, School of Basic Medical Sciences, Anhui Medical University, Hefei, China; 2 Department of Pharmacology, School of Pharmaceutical Sciences, Anhui Medical University, Hefei, China

**Keywords:** autoimmune diseases, epigenetics, immune regulation, m6A, RNA methylation, therapeutic strategies

## Abstract

Immune-mediated diseases arise from intricate interactions among genetic, environmental, and epigenetic factors that disrupt immune homeostasis. In recent years, epigenetic mechanisms have been widely explored as critical factors in autoreactivity. Among these modifications, N6-methyladenosine (m6A) RNA methylation stands out as a pivotal post-transcriptional regulator of immune cell function and autoimmune diseases (ADs) progression. This review outlines m6A regulation in immune microenvironments and its dual role in maintaining tolerance and promoting inflammation. This study highlights how m6A regulators, including writers (METTL3/14), erasers (FTO and ALKBH5), and readers (YTHDF1–3 and IGF2BP3), orchestrate immune cell dysfunction across systemic (systemic lupus erythematosus (SLE), rheumatoid arthritis (RA), psoriasis) and organ-specific (multiple sclerosis (MS), inflammatory bowel disease (IBD), type 1 diabetes mellitus (T1DM), and autoimmune thyroid disease (AITD) ADs, revealing disease-specific epitranscriptomic regulatory patterns. Critically, we highlight recent therapeutic breakthroughs targeting m6A regulators, including METTL3 inhibition (STM2457) for Th17-driven MS and RA synovitis, ALKBH5 modulation (ALK-04) to mitigate psoriasis flares and neuroinflammation, FTO-targeting small molecules (Rhein) to prevent RA-associated bone erosion, and IGF2BP3 blockade (triptolide) to suppress RA fibroblast activity. Despite their promise, key challenges persist, including stage-specific effects (early vs. chronic), rare immune subset targeting (MDSCs in AIH), and concerns about the long-term safety of epitranscriptomic drugs. Future studies must address m6A dynamics in immune crosstalk to advance precision medicine strategies, particularly through combinatorial approaches with existing JAK inhibitors or checkpoint modulators.

## Graphical Abstract

**Figure:**
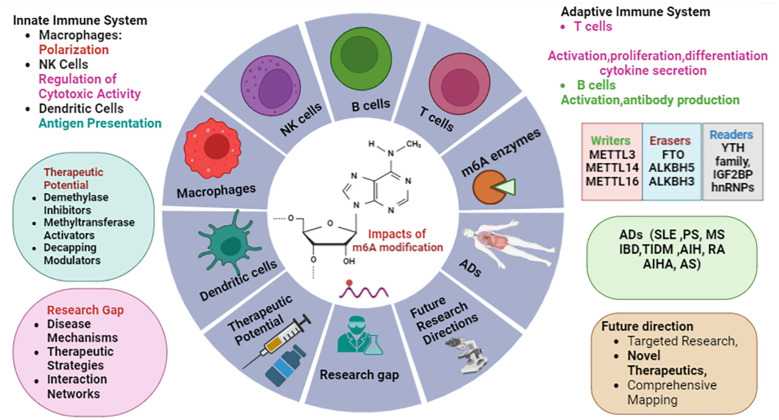

## 1 Introduction

ADs which affect nearly 10% of the global population, arise from a complex interplay of genetic, environmental, and immunological factors that disrupt immune tolerance (1–3). Traditionally, epigenetic research has focused on DNA methylation and histone modifications; however, in recent years, there has been a paradigm shift towards RNA epigenetics (4, 5). To date, over 100 distinct RNA modifications, including mRNAs, rRNAs, tRNAs, snRNAs, miRNAs, and long noncoding RNAs (lncRNAs), have been identified across various RNA species (6). m6A is the most abundant internal modification in mRNAs, with other common modifications including N1-methyladenosine (m1A), 5-methylcytosine (m5C), N7-methylguanosine (m7G), RNA cap methylations, pseudouridine, and uridylation (7, 8). m6A typically follows a consensus pattern, with 1–2 m6A residues per 1000 nucleotides, mainly in RRACH sequences (where R symbolizes G or A, and H symbolizes A, C, or U) (9).

m6A is crucial for RNA metabolism and regulates premRNA splicing, cap-independent translation, mRNA decay, and miRNA synthesis in ADs. m6A is a key regulator of immune-cell function and immune homeostasis, influencing immune tolerance and inflammatory responses through the coordinated actions of its writers, erasers, and readers. Although dysregulated m6A signaling has been implicated in aberrant immune activation and ADs pathogenesis, its regulators often exert distinct and sometimes contradictory effects across immune-cell lineages, tissue micro-environments, disease stages, and inflammatory contexts (10). For example, METTL3 may promote inflammation in some immune cells while supporting immune tolerance in others. These context-dependent effects complicate our understanding of m6A-mediated regulation in autoimmunity and highlight the need for an integrated framework linking epitranscriptomic regulation to autoimmune pathogenesis.

Unlike previous reviews that have summarized the molecular biology of m6A or its role in individual immune disorders, a comprehensive synthesis integrating immune-cell-specific mechanisms with comparative analyses across systemic and organ-specific ADs remains limited (4, 5). This review fills that gap by providing a unified framework for understanding how m6A dysregulation contributes to autoimmunity. Specifically, we summarize the m6A regulatory complex (writers, erasers, and readers) with emphasis on immune-relevant mechanisms; compares m6A-mediated regulation across innate immune cells (DCs, macrophages, NK cells) and adaptive immune cells (T cells, B cells). We provide a disease-by-disease analysis of m6A dysregulation in systemic ADs (SLE, RA, psoriasis) and organ-specific ADs (T1DM, AITD, MS, IBD), highlighting shared and disease-specific patterns. In addition, we critically evaluate emerging therapeutic strategies targeting m6A regulators, including METTL3 inhibitors (e.g., STM2457), FTO modulators (e.g., rhein, DAC51), ALKBH5 inhibitors (e.g., ALK-04), and reader protein blockade (e.g., triptolide). We further discuss the clinical unmet needs driving m6A-targeted therapies and the potential interplay between m6A regulation and immune checkpoint-based immunotherapy. Finally, we highlight unresolved controversies, including the paradoxical, context-dependent effects of METTL3, limitations of the M1/M2 macrophage paradigm, and key knowledge gaps that remain to be addressed for clinical translation.

## 2 m6A regulatory complex

**Figure 1:**
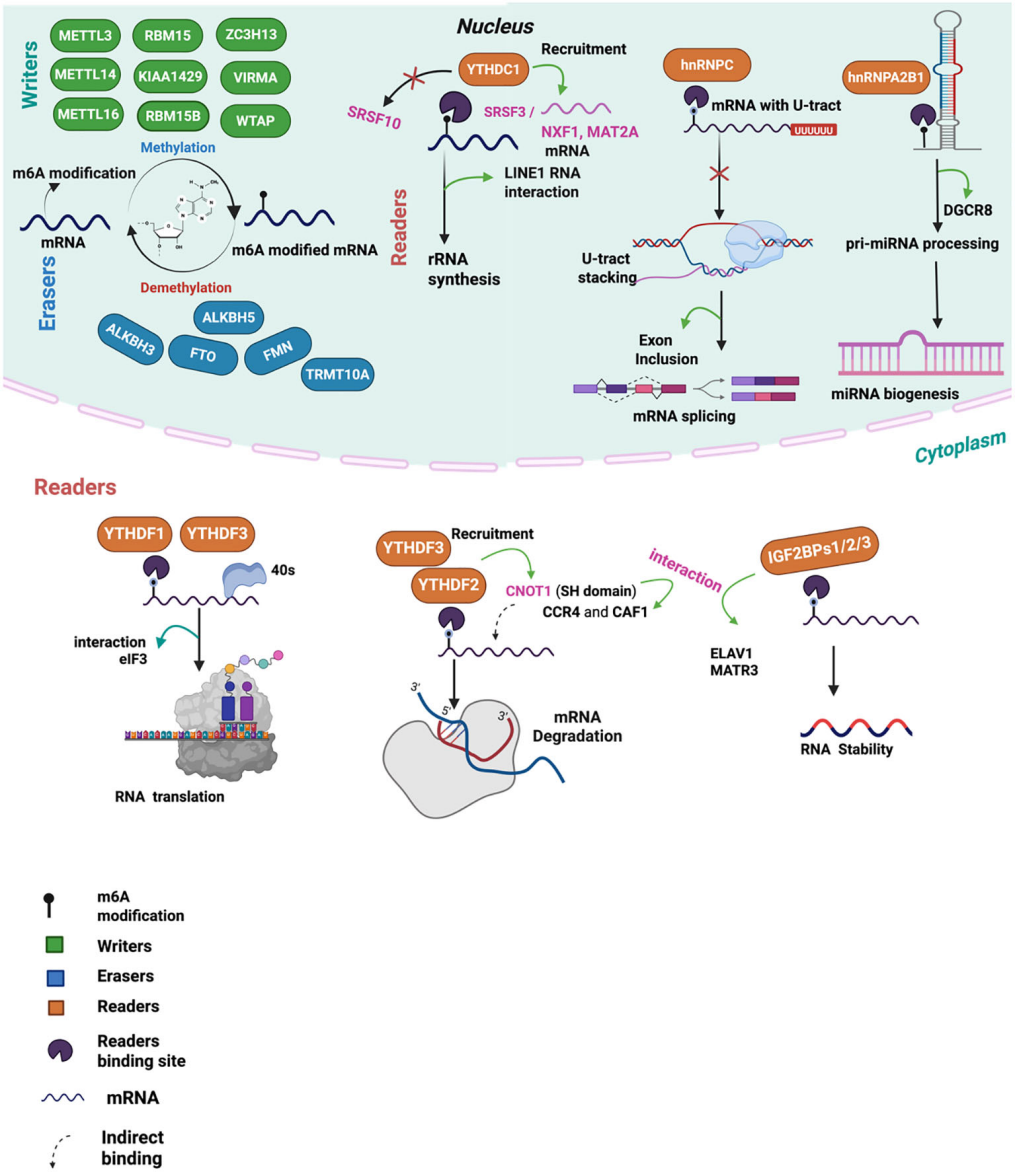
m6A regulation machinery. m6A is added by writers (METTL3, METTL14, METTL16, WTAP, VIRMA, RBM15/15B, ZC3H13) and removed by erasers (FTO, ALKBH5). Readers (YTHDFs, YTHDCs, IGF2BPs, HNRNPC/A2B1) regulate splicing, export, translation, stability, and decay.

m6A is regulated by writers (methylates 3’ UTR RRACH sites), erasers (remove S-adenosylmethionine, regulate gene expression/cell fate), and readers (control m6A function, affecting RNA binding/structure) (11). Dysregulation contributes to disease (**Figure 1**; **Table 1**).

**Table 1:** Target RNAs and functions of m6A regulatory proteins.

Type	Enzymes	Target RNAs	Functions	References
Writers	METTL3, METTL14	mRNAs lncRNAs miRNAs	m6A deposition on nuclear RNA in mammalian cells.	(12–18)
	METTL5-TRMT112	rRNA	Responsible for m6A modification of 18S rRNA	(19)
	METTL16	lncRNA snRNA mRNA	Regulation of the dynamic homeostasis of SAM.	(20)
	WTAP	mRNA	METTL3-METTL14 complex localizes to nuclear patches enriched with pre-mRNA processing factors.	(21)
	ZC3H13	mRNA	Nuclear localization of the ZC3H13-WTAP-Virilizer-Hakai complex. Regulation of self-renewal of MESC.	(22)
	RBM15/15B	lncRNA mRNA	Recruiting MTC to specific sites in RNA; The suppression of one X chromosome in female cells.	(23)
	HAKAI	mRNA	mRNA methylation in Arabidopsis thaliana.	(24, 25)
	VIRMA/KIAA1429	mRNA	Recruitment of catalytic core components METTL3/METTL14/WTAP.	(21)
Erasers	FTO	mRNA tRNA snRNA	Oxidation of m6A to produce hm6A for further oxidation to f6A.	(26–31)
	ALKBH5	mRNA	Affects mRNA export from nuclear patches, RNA metabolism, and the assembly of mRNA processing factors.	(32, 33)
	ALKBH3	tRNA	Improvement of protein translation efficiency.	(34, 35)
	FMN	mRNA	Synthetic m6A demethylase modulate gene expression.	(36)
	TRMT10A	mRNA	Enhancement of m6A demethylase activity of FTO.	(37)
Readers	YTHDC1	mRNA	Mediation of the export of methylated mRNA from the nucleus to the cytoplasm.	(38–41)
	hnRNPC	mRNA	Affects the abundance and selective splicing of target mRNAs.	(42, 43)
	hnRNPG	mRNA	Altering the expression and variable splicing pattern of target mRNA.	(44)
	YTHDF1	mRNA	Promoting more efficient translations.	(45–47)
	YTHDF2	mRNA	Reduced messenger stability; degradation, affects the decay of methylated mRNA.	(48, 49)
	YTHDF3	mRNA	Promote translation.	(46)
	YTHDC2	mRNA	Spermatogenesis process.	(47)
	IGF2BP1/2/3	mRNA	Promotes the stabilization and storage of its target mRNAs and affects mRNA export.	(49)
	eIF3	mRNA	Recruitment of the 43 S complex to initiate translation.	(50)
	hnRNPA2B1	pri-miRNA	Nuclear pri-miRNA processing and selective splicing.	(51)
	PRRC2A	mRNA	Maintains Treg stability by binding m6A-modified STAT3 mRNA.	(52)
	FMRP	mRNA	Stabilize FOXP3 mRNA to preserve Treg suppressive function	(53)

### 2.1 m6A writers: mechanistic insights and regulatory roles

The m6A methyltransferase complex is composed of METTL3, METTL5, METTL14, METTL16, RBM15, RBM15B, ZC3H13, VIRMA (KIAA1429) and WTAP, and cofactors catalyze RNA methylation in the nucleus and cytoplasm (12). METTL3 is responsible for more than 95% of m6A synthesis in mRNAs contains a SAM-binding domain and a DPPW motif (Asp-Pro-Pro-Trp), facilitating its stable 1: heterodimerization with METTL14, which enhances RNA substrate specificity (13). METTL3-mediated m6A stabilizes TLR4 mRNA, promoting neutrophil activation via TLR4/NF-κB (14). METTL3/RBM15-mediated m6A on KDM6B stabilizes transcripts and enhances IFN-γ–driven JAK1–STAT1 inflammatory signaling (15). Notably, METTL3 SUMOylation suppresses its methyltransferase activity without affecting protein stability, or METTL14/WTAP interaction, representing a potential checkpoint in inflammation regulatory (16). The RGG motif of METTL14 (Arg-Gly-Gly) interacts with RNA polymerase II at replication-stress checkpoints, linking DNA damage to m6A deposition. Its SAM-binding and EPPL (Glu-Pro-Pro-Leu) promote transcription complex interactions (17). The METTL3–METTL14 heterodimer is assembled cotranslationally via the chaperonin CCT4; disruption by M14P1 reduces m6A deposition and proliferation, suggesting therapeutic potential (18). METTL5 forms a heterodimer with TRMT112 to catalyze m6A at A1832 on 18S rRNA, aided by ZCCHC4 (19). METTL16 methylates lncRNAs and U6 snRNA A43 at the “UAC(m6A)GAGAA” motif. Its N-terminal domain regulates MAT2A splicing for SAM homeostasis (20). WTAP-VIRMA restricts METTL3-METTL14 dsDNA binding and promotes RNA-specific m6A methylation (21). Nuclear localization signals in METTL3 and WTAP enable nuclear entry, regulating m6A sites under stress, while WTAP analogs Mum2 and FIP37 enhance this via METTL3 interaction (54, 55). ZC3H13 promotes m6A methylation by facilitating the nuclear localization of the ZC3H13-WTAP-Virilizer-Hakai complex (22). RBM15/15B regulate XIST m6A methylation and gene silencing (23), while E3 ligase HAKAI contributes to m6A modification and may catalyze m6A (24, 25).

### 2.2 m6A erasers: mechanistic insights and regulatory roles

The m6A modification is reversible. FTO (discovered in 2011) and ALKBH5 (discovered in 2013) are the primary demethylases “erasers” that remove m6A marks from RNA (26, 27). Both are Fe(II)/α-ketoglutarate-dependent demethylases and share AlkB domains containing HXDXnH/RXXXXXR motifs (28, 29). FTO’s C-terminal loop undergoes substrate-induced conformational rearrangement, enabling selective recognition of m6A-mRNA or m6A-U6 snRNA at the Fe(II)/α-ketoglutarate catalytic centre, supporting tissue-specific activity in brain and adipose tissue (30). FTO-mediated RNA demethylation reveals a complex, multistep pathway characterized by the oxidation of m6A to hm6A, then f6A, and finally converts back to adenosine (31). ALKBH5, predominantly expressed in the testis but also has significant roles in immune regulation. It oxidatively reverses m6A by converting the nitrogen-linked methyl group to a hydroxymethyl intermediate that spontaneously releases formaldehyde and regulates mRNA export and ribosome biogenesis in nuclear speckles (32). ALKBH5 deficiency exacerbates experimental autoimmune encephalomyelitis via m6A-sustained IFNG mRNA and Th1/Th17 polarization (33). ALKBH3 preferentially targets tRNA over mRNA or rRNA, demethylating m1A, m3C, and m6A to enhance translation but lacks mRNA m6A eraser activity (34). ALKBH3 mediates RNA m1A demethylation through a Fe(II)/α-ketoglutarate-dependent oxidative mechanism, with key catalytic residues Asp194 and Thr133 stabilizing the flipped-out m1A base to ensure precise substrate recognition and efficient methyl group removal (35). FMN acts as a potent synthetic m6A demethylase (36). TRMT10A enhances substrate selectivity and functions synergistically with FTO in RNA modification (37).

### 2.3 m6A readers: mechanistic insights and regulatory roles

m6A-modified RNA is recognized by “reader” proteins that determine the fate of target transcripts. Readers are classified as nuclear (YTHDC1, hnRNPC, hnRNPA2B, hnRNPG) and cytoplasmic (YTHDF1-3, YTHDC2, IGF2BP1-3), modulating splicing, export, translation, stability, and degradation (56–58). YTHDC1 regulates alternative splicing by recruiting SRSF3, inhibiting SRSF10, and controlling NXF1 and MAT2A mRNA, while facilitating rRNA synthesis via Long Interspersed Nuclear Element-1 (LINE1) RNA (38, 39). In autoimmunity, YTHDC1 regulates macrophage inflammation via m6A-dependent Beclin-1 mRNA stabilization and NF-κB inhibition (40). It also binds METTL3-methylated m6A on lncRNA MEG3 to regulate early autoimmune inflammation (41). hnRNPC disrupts U-tract stacking to increase RNA accessibility, binding splice sites in pri-mRNA to regulate RNAPII and promote exon inclusion (42, 43). hnRNPA2B1, with multiple RRM domains, binds to RGAC motifs and the C-terminal segment of m6A-modified RNAs, promoting pri-miRNA processing and splicing via DGCR8 (59). hnRNPG binds m6A-modified nascent mRNAs and phosphorylated RNAPII CTDs through the Arg-Gly-Gly (RGG) motif, co-transcribing with RNAPII to regulate selective splicing (44).

YTHDF1 binds CD80 mRNA via m6A-eIF3 complex, enhancing neoantigen degradation in DC and worsening antigen presentation in RA (45), while enhancing GBP4-mediated M1 polarization in acute lung injury (60). YTHDF2 facilitates mRNA degradation binding to the SH domain of CNOT1 and recruits CCR4-NOT and HRSP12/RNaseP-MRP to degrade SOCS1 transcripts, sustaining JAK/STAT hyperactivity in RA fibroblasts (48). YTHDF3 has dual functions, enhancing circRNA translation via YTHDF1 and promoting RNA degradation through YTHDF2 (46). YTHDC2 promotes translation and ASH1L stabilization, limiting IL-17/IL-23R and Th17 responses (47). IGF2BP2 binds to GG(m6A)C motifs to stabilizes key mRNAs (GPX4, SOX2, VEGFA, CSF2/IL-6) with ELAVL1 and HOTAIR, promoting inflammation, EMT, angiogenesis, and therapy resistance (49). eIF3 binds the m6A-modified 5′UTR of IFNB1 mRNA to promote cap-independent translation through the 43S complex, driving interferon production in plasmacytoid DC without eIF4E (50). PRRC2A uses its GRE domain to bind m6A-modified STAT3 mRNA, preserving Treg stability and limiting colitis (51). FMRP and YTHDF2 protect FOXP3 mRNA from m6A decay, ensuring Treg suppressive function (61).

In summary, reader proteins act as the ultimate molecular switch that determines the fate of m6A-modified transcripts. YTHDF1 promotes translation and stability, YTHDF2 facilitates mRNA degradation, and IGF2BP proteins stabilize target transcripts. Thus, whether an inflammatory program is amplified or suppressed depends on which reader protein binds to the m6A-modified transcript, providing a mechanistic basis for the selective regulation of inflammatory responses in autoimmune diseases.

## 3 m6A modifications in immune cells

m6A regulate innate APCs (DCs, macrophages), adaptive immune cells (T/B cells), and NK cells, influencing cytokine production and immune responses (**Table 2**).

**Table 2:** Comparative overview of immune cells in ADs.

Immune cell type	m6A regulators/ADs associated	Mechanistic impact	References
DCs	METTL3↑ in RA, SLE	Enhances MHC-II, CD80/CD86, IL-12 via TLR4/NF-κB, promoting T cell activation.	(62, 63)
	YTHDF1↑ in MS	Stabilizes lysosomal proteases, boosts antigen cross-presentation.	(52, 64)
	YTHDF2↑ in IBD	Degrades Lnc-Dpf3, regulates antigen presentation & migration.	(53, 65)
	FTO↓ in RA	Alters glycolytic metabolism, modulates inflammatory cytokine output.	(63, 66)
**Macrophages (M1)** **Macrophages (M2)**	METTL3 ↑ in RA, SLE	Promotes pro-inflammatory polarization via STAT1/IRF5 mRNA stabilization.	(67–69)
	METTL14 ↑ in RA	Enhances inflammatory gene translation through m6A-YTHDF1 axis.	(66, 70)
	YTHDF2 ↑ in IBD	Destabilizes SOCS1/SOCS3 mRNA, amplifying NF-κB signaling.	(71, 72)
	FTO↑ in SLE	Supports anti-inflammatory phenotype by demethylating PPARγ mRNA.	(73–75)
	ALKBH5↑ in RA	Stabilizes SOCS2 mRNA, limiting STAT3 activity.	(76)
**NK cells**	METTL3↓ in SLE	Reduces cytotoxicity via impaired mTORC1-EOMES axis.	(77, 78)
	YTHDF2 ↑ in MS	Regulates STAT5-IL-15 signaling, controls proliferation.	(79)
**T Cells** **(Th1/Th17)** **Th17** **CD4+ Tregs** **CD8+ T Cells**	METTL3 ↑ in RA, MS	Promotes pathogenic Th1/Th17 differentiation via T-bet/RORγt mRNA stability.	(10, 80)
	METTL14 ↑ in MS, IBD	Supports GM-CSF expression and encephalitogenicity.	(10, 62, 81–96)
	PRRC2A ↑in IBD	Binds m6A-STAT3 mRNA, preserves Treg stability in colitis.	(51)
	FMRP ↑ in IBD	Protects FOXP3 mRNA from m6A decay.	(61)
	YTHDF2 ↑ in IBD	Prevents FOXP3 mRNA degradation, sustaining suppressive function.	(84)
	METTL3↑ in RA, MS	Supports effector differentiation via IL-7/STAT5 axis.	(80, 97)
	YTHDF1↑ in MS	Enhances granzyme B translation.	(98)
**B cells**	METTL3↑ in SLE	Promotes plasma cell differentiation through BLIMP1 mRNA stabilization	(87, 88)
	YTHDF2↑ in SLE	Regulates BCR signaling thresholds.	(95)
	ALKBH5↓in RA	Reduces AID mRNA stability, affecting class-switch recombination.	(83)

DCs, dendritic cells; M1 macrophages, classically activated macrophages; M2 macrophages, alternatively activated macrophages; NK cells, natural killer cells; T cells, T lymphocytes; Th1/Th17, T helper 1/T helper 17 cells; Th17, T helper 17 cells; CD4^+^ Tregs, CD4^+^ regulatory T cells; CD8^+^ T cells, CD8^+^ cytotoxic T lymphocytes. An upward arrow (↑) indicates increased expression of m6A regulators, whereas a downward arrow (↓) indicates decreased expression of m6A regulators.

### 3.1 Antigen-presenting cells (APCs) and m6A regulation

#### 3.1.1 Dendritic cells (DCs): m6A and antigen presentation

m6A RNA methylation critically regulates DCs activation and function (99). METTL3 knockdown reduces m6A on MHCII, co-stimulatory molecules (CD80, CD86, TIRAP), and cytokines (IFN-γ, IL-12), impairing T cell activation and DC-mediated immune tolerance through the TLR4/NF-κB pathway (100). Beyond this, METTL3 regulates miRNA processing, chromatin accessibility, and metabolic pathways (mTOR/HIF-1α), supporting DC activation (45, 101). In contrast, METTL3/14 hyperactivation and autoantibody-triggered TLR signaling in DCs increase IL-6, TNF-α, IFN-α in lupus, RA, and SLE, disrupting CD4+T, CD8+T cells and B cells (75). METTL3 drives cross-priming in cDC1s, while pDCs rely on m6A for regulation of type I interferon (102).

**Figure 2:**
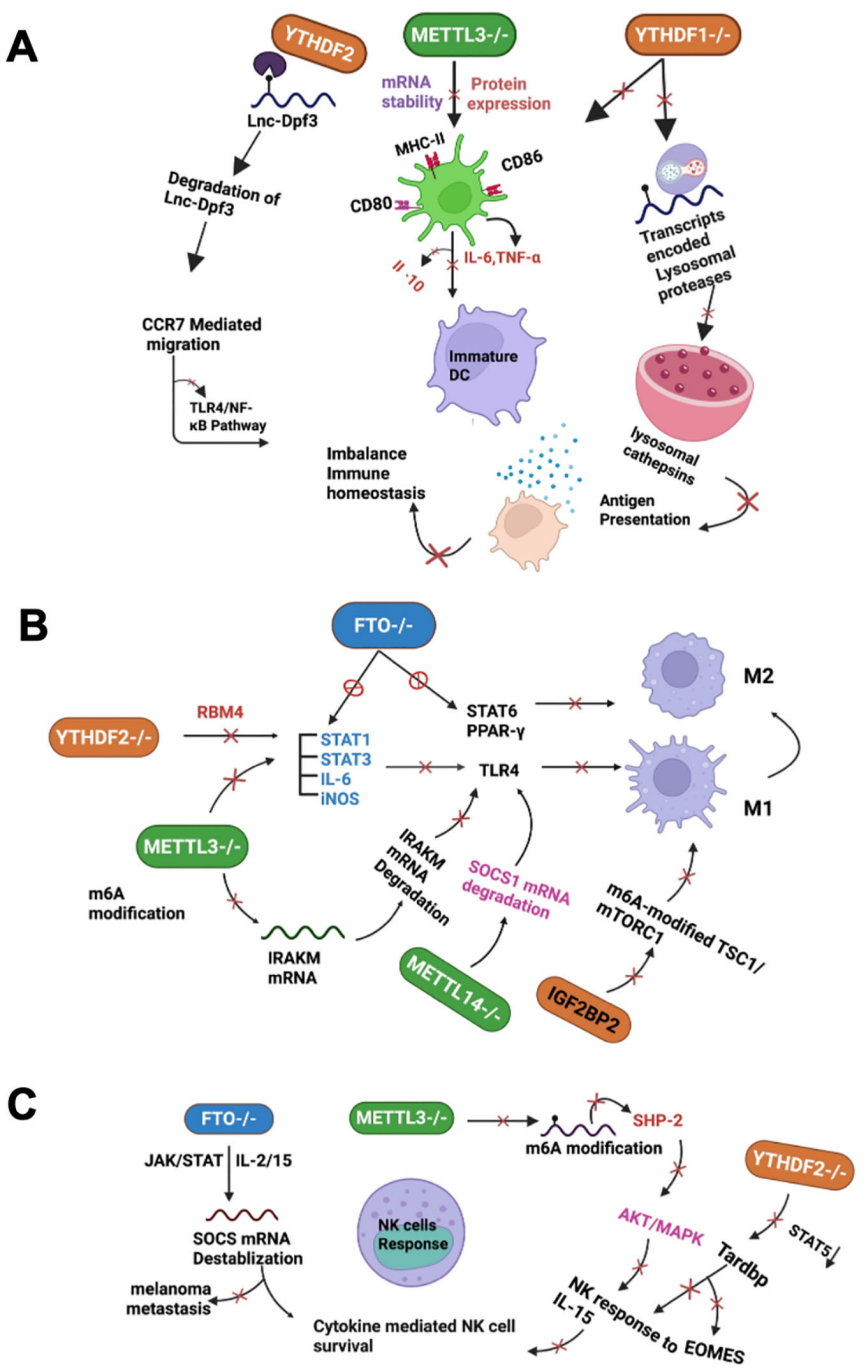
Impact of m6A RNA modification on innate immune system regulation. **(A)** DC; YTHDF1 loss impairs lysosomal cathepsin translation and DC maturation. METTL3 loss destabilizes CD80, CD86, TIRAP, and MHC II, altering IL-10, IL-6, and TNF-α. YTHDF2 loss enhances lnc-Dpf3 degradation and CCR7-mediated migration.**(B)** Macrophages; METTL3 regulates STAT1/IRAKM, controlling TLR4 signaling and M1/M2 balance; FTO regulates STAT1, STAT6, and PPAR-γ via NF-κB; IGF2BP2 promotes TSC1/mTORC1-mediated polarization. **(C)** NK; METTL3, FTO, and YTHDF2 regulate SHP-2/AKT-MAPK, JAK/STAT, SOCS, EOMES, and STAT5, controlling NK activation and antitumor responses.

YTHDF1deficiency enhances DCs cross-presentation and strengthens their cross-priming capacity, leading to an increased CD8+T cell–mediated immune response. This reflects improved antigen availability for MHC presentation due to reduced YTHDF1-dependent lysosomal cathepsin translation and decreased antigen degradation (52). YTHDF2 induces Lnc-Dpf3 degradation by binding its m6A sites. Lnc-Dpf3 controls antigen presentation, T cell activation, cytokine production, CCR7 migration, and inhibits glycolytic genes via HIF-1α (53). YTHDF2 upregulation by SPI-1 degrades m6A-modified Mfng, Aph-1b, and Aph-1c transcripts in irradiated DCs, inhibiting Notch signaling, MHC-I expression, CD8+T-cell priming, and radiotherapy immunity (**Figure 2A**) (103).

However, the role of METTL3 in DCs remains incompletely understood. One study reports that METTL3 deficiency impairs DC activation and CD4+T-cell priming (100), while another shows that METTL3 hyperactivation drives inflammation in lupus and RA (75). This opposing effect is not a true contradiction but reflects immune microenvironmental regulation. METTL3 acts as a rheostat: low-to-moderate activity supports DC-mediated immune tolerance, whereas excessive activation drives a pro-inflammatory phenotype. Immunological regulation is influenced by disease stage, (early vs chronic), inflammatory milieu (presence of autoantibodies or TLR ligands), and DC subset (cDC1 vs cDC2 vs pDC), indicating that METTL3 is neither inherently pro-inflammatory nor tolerogenic its function is determined by the cellular and tissue environment. YTHDF1 also shows a dual and debated role in DCs, as it regulates antigen processing through lysosomal protease translation and modulates CD8+T cell priming by affecting antigen degradation in different cellular and immune contexts, indicating subset-specific effects (cDC1 vs pDC). Future m6A-/CLIP-seq studies should define METTL3-regulated transcriptomes in tolerogenic vs. immunogenic DC subsets underlying distinct autoimmune pathologies.

#### 3.1.2 Macrophages: m6A in pro-and anti-inflammatory polarization

m6A regulates macrophage activation and polarization into pro-inflammatory M1 (antigen presentation, T-cell activation) and anti-inflammatory M2 (immunosuppressive), influencing immune responses in various ADs (**Figure 2B**) (104). Loss of METTL3 in macrophages reduces m6A on STAT1 and IRAKM, inhibiting TLR4 signaling and promoting M1-to-M2 polarization. In RA PBMCs, elevated METTL3 correlates with CRP/ESR and may suppress NF-κB–mediated inflammation, and promotes M1 polarization by regulating IL-6, iNOS, SOCS, and TNF-α (67–69). METTL14 is upregulated in M1 macrophages, its knockdown restores M2 gene expression while suppressing TNF-α and IL-6 secretion, identifying it as a regulator of macrophage-driven joint inflammation in RA (70).

Silencing FTO reduces STAT1 expression in M1 macrophages and STAT6/PPAR-γ in M2 macrophages, inhibiting polarization and NF-κB signaling by decreasing STAT phosphorylation and destabilizing target mRNAs (73). FTO demethylates m6A on miR-31-5p, reducing its stability and enhancing P2RX7/p38 MAPK signaling to promote M1 polarization in autoimmune dry eye (74). ALKBH5 promotes CCL5 mRNA stability via m6A demethylation, enhancing macrophage dysfunction and infiltration of IFN-γ–producing CD8+T cells through the CCL5-CCR5-autophagy pathway during atherosclerosis (76). YTHDF2 deficiency prevents STAT1 mRNA degradation, inhibiting macrophage activation and M1 polarization (71). IGF2BP2 regulates macrophage activation via m6A-modified TSC1/mTORC1; its loss reduces IL-4 response, promoting M1 polarization (105, 106).

Collectively, these findings suggest that macrophage polarization in ADs is highly dynamic and cannot be strictly confined to the classical M1/M2 paradigm, as intermediate and mixed phenotypes are frequently observed *in vivo* highlighting, a key limitation of binary classification models. First, human autoimmune tissues (RA, MS, IBD) contain macrophages co-expressing M1 and M2 markers rather than distinct subsets. Second, macrophage polarization is highly dynamic and reversible; the same macrophage can transition between phenotypes depending on disease stage, tissue microenvironment, and inflammatory cues. Third, *in vitro* M1/M2 models do not fully reflect *in vivo* macrophage heterogeneity, indicating a need for a more refined framework (107).

### 3.2 Innate natural killer (NK) cells: m6A and cytotoxicity

NK cells, key to innate immunity, interact with macrophages, DCs, T cells, and endothelial cells to modulate immunity in cancer, autoimmunity, and inflammation (10). METTL3 deficiency reduces SHP-2 expression, impairing AKT-MAPK signaling and NK-cell responsiveness to IL-15 (**Figure 2C**) (77, 78). METTL14-mediated m6A modification at the 3’ UTR of Gzmb mRNA prevents decay, stabilizing granzyme B expression, while METTL3 enhances NKG7 and CXCR6 translation to promote cytotoxicity, distinct from its anti-inflammatory role in Tregs (108). FTO deletion elevates m6A on SOCS1/3 mRNAs, accelerating their decay, amplifying JAK/STAT signaling and enhancing NK-mediated control of melanoma and leukemia (109). FTO and ALKBH5 regulate NKG2D mRNA demethylation in NK cells, controlling antitumor immune responses (110). YTHDF2 regulates NK-cell homeostasis by degrading EOMES/Tardbp mRNAs, enhancing STAT5 signaling, survival, proliferation, and cytotoxicity; it also stabilizes KLRD1/CD107A in NK cells but promotes mRNA decay in monocytes (79).

METTL3 promotes NK-cell cytotoxicity in some contexts yet plays an anti-inflammatory role in Tregs, indicating cell-type-specific effects and highlighting the need for multi-omics studies to evaluate m6A-targeted immune modulation.

### 3.3 Adaptive immune cells: m6A in T and B cell regulation

#### 3.3.1 T cells: m6A as a modulator of autoimmunity

**Figure 3:**
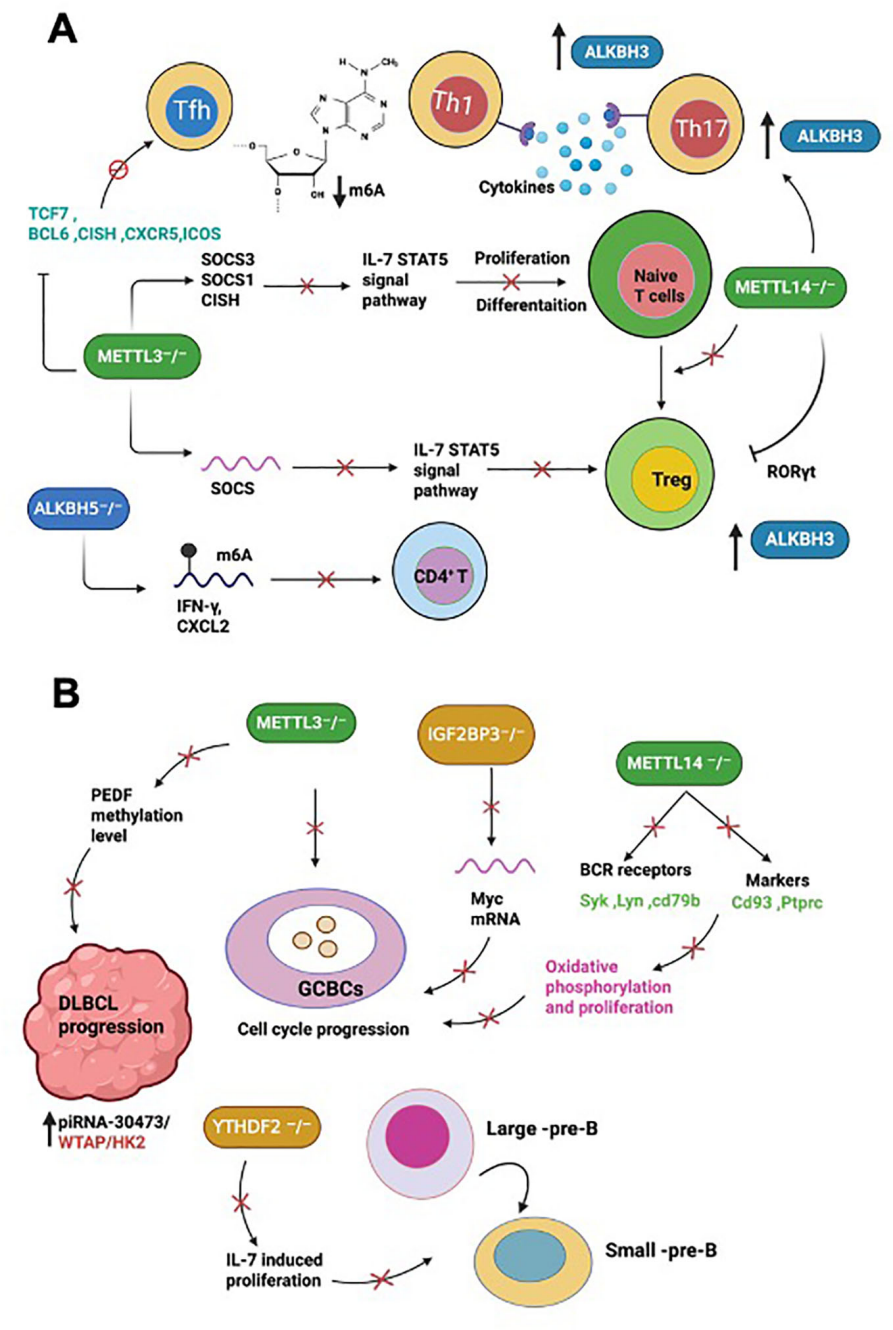
m6A RNA modification in the adaptive immune system. **(A)** CD4+T cells/Tregs; METTL3 loss increases SOCS (CISH, SOCS1, SOCS3), suppressing IL-7/STAT5 and T cell differentiation. METTL3 loss in Tregs reduces IL-2/STAT5 and suppressive function. METTL14 loss increases Th1/Th17 cytokines and reduces Treg differentiation via RORγt. ALKBH5 loss reduces IFN-γ, CXCL2, and T cell responses. **(B)** B cells; METTL3 loss reduces PEDF and Myc, impairing proliferation, oxidative phosphorylation, and cell cycle. YTHDF2 loss disrupts B cell development; METTL14 loss impairs IL-7–driven pre-B transition via BCR downregulation.

CD4+T and CD8+T cells are the two main categories within T cells. m6A RNA methylation plays a central role in controlling T-cell differentiation, activation, and immune homeostasis (97). METTL3-mediated m6A at the 3’-end of SOCS2 transcripts, recognized by YTHDF2, limits SOCS1, SOCS3, and CISH, promoting IL-7/IL-2 STAT5 activation (**Figure 3A**). METTL3 deficiency elevates SOCS mRNAs, suppressing STAT5 and impairing T cell differentiation and Treg function (80). In addition, METTL3 modulate Tfh differentiation by stabilizing TCF7 mRNA and sustaining key markers (CXCR5, BCL6, CISH, ICOS) essential for germinal center and humoral immunity (111). In a colitis model, METTL14-deficient T cells promoted inflammation via enhanced Th1/Th17 cytokines and impaired Treg differentiation. Reduced RORγt in Tregs aggravated IBD and promotes transplant rejection by disrupting m6A-YTHDF2-dependent stabilization of immunoregulatory Sema4D mRNA, underscoring METTL14’s role in T cell-mediated immune homeostasis (81). FTO enhances CD8+T cell survival by demethylating Fas mRNA, inhibiting IGF2BP3 stabilization and reducing Fas-mediated apoptosis (82). FTO deficiency disrupts Th1 differentiation and immunity by lowering T-bet and IFN-γ, in CD4+T cells (10). ALKBH5 mRNA levels increase in activated Th1, Th2, Th17, and Treg cells compared to naïve CD4+T cells. ALKBH5 reduces MDSC immunosuppression by demethylating Arg-1 mRNA, lowering its stability and impairing arginase 1-mediated T cell suppression (83). YTHDF2 promotes anti-tumor activity in CD8+T cells by sustaining IKZF1/3-driven transcription and mitochondrial integrity, supporting effective immunotherapy (84). Despite these insights, controversy remains regarding METTL3 function in T cells. One study reported that Loss of METTL3 impairs Treg differentiation and function via the SOCS/STAT5 pathway, whereas another study found that METTL3 also promotes Tfh differentiation through TCF7 stabilization.This suggests that METTL3 function is context-dependent, but the underlying mechanisms remain unknown.

#### 3.3.2 B Cells: m6A in antibody production and autoantibody generation

B cells mediate humoral immunity, using B cell receptors (BCRs) to recognize antigens and mature into plasma cells producing antibodies (85, 86). METTL3-mediated m6A stabilizes EZH2 mRNA, promoting B cell survival and proliferation in primary Sjögren’s syndrome autoimmunity (87). METTL3-dependent m6A regulation in B cells promotes regulatory B-cell (Breg) differentiation and stabilizes immune homeostasis, thereby suppressing pro-inflammatory cytokine production and limiting neuroinflammation (88). METTL14 deficiency reduces m6A, impairing IL-7-driven transition from large to small pre-B cells by disrupting B cell markers, BCR signaling, and proliferation control. Its methylation degrades Lax1 and Tipe2 to promote follicular B cells, while with METTL3, it supports YTHDF2-dependent IL-7-induced pro-B differentiation (89). DLBCL, a common lymphoid cancer with rapid B-cell growth, shows increased METTL3 and m6A methylation that promotes progression via PEDF transcript methylation (**Figure 3B**). Silencing METTL3 disrupts this methylation, decreasing DLBCL proliferation (90). METTL3 deficiency in GCBCs slows the cell cycle, reduces proliferation and oxidative phosphorylation gene expression, impairing immune response development (91). METTL3 and IGF2BP3 stabilize Myc mRNA and protein, regulating cell cycle and metabolism in GCBCs (92). GCBCs rely on mitochondrial and peroxisomal fatty acid oxidation for respiration, with YTHDF2 indirectly regulating oxidative phosphorylation genes, emphasizing metabolic control in GCBC function (93). piRNA-30473 promotes WTAP-mediated m6A of HK2 mRNA, elevating HK2 expression and driving DLBCL progression (94).

YTHDF2 deletion impairs pro-B to large pre-B transition by reducing chromatin accessibility at transcription factors and BCR recombination genes essential for B cell development; it promotes plasma cell differentiation and CD138+ survival, whereas EIF3K and EIF3L negatively regulate this process (95). RBM15 loss reduces peripheral B cells and delays early B cell differentiation without affecting IgM/IgD ratios, suggesting limited germinal center B cell involvement (96). It remains unknown how m6A regulates the breach of B cell tolerance and autoantibody affinity maturation.

## 4 m6A dynamics in systemic and organ-specific ADs

m6A contributes to immune dysregulation in systemic (SLE,RA,Ps) and organ-specific ADs (T1DM, AITD, MS, and IBD), with emerging evidence supporting its role in MG, Systemic Sclerosis, Celiac Disease, Uveitis, and AS (10).

### 4.1 Epitranscriptomics impact of m6A on systemic ADs

#### 4.1.1 m6A methylation in systemic lupus erythematosus (SLE)

SLE, involving immune dysregulation and loss of T/B cell self-tolerance, leads to kidney, joint, skin, and nervous system inflammation in alternating flares and remissions (62). Luo et al. first reported the downregulation of METTL14, ALKBH5, and YTHDF2 in SLE PBMCs, correlating with lower immune cells, heightened inflammation, and disease worsening (112, 113). In SLE, METTL3 downregulation in CD4+ T cells correlates with disease activity and promotes Treg differentiation through NF-κB modulation and Foxp3 stabilization in cGVHD models (75). Upregulated METTL3 enhances m6A modification of IRF4, driving plasma cell-mediated renal damage in SLE (114). Zhao et al. found glomerular METTL3, WTAP, YTHDC2, YTHDF1, FMR1, and FTO correlate with GFR and NK cell activation, predicting lupus nephritis prognosis (115). In SLE, METTL3 regulates PAX5 via m6A modification, while PAX5 reciprocally controls METTL3, forming a feedback loop driving B-cell hyperreactivity and autoimmunity (116). In SLE, m6A dysregulation destabilizes FoxO1 mRNA in monocytic MDSCs, impairing their immunosuppressive function and promoting B-cell hyperactivation and disease progression (117). In SLE, lower ALKBH5 in PBMCs and T cells correlates with higher anti-dsDNA, disease activity, C3, and CRP, while increased ALKBH5 promotes apoptosis and suppresses proliferation (112). In SLE, m6A methylation of mitochondrial NADH dehydrogenase 6 impairs CD4+T cell oxidative phosphorylation, increasing ROS, reducing ATP, and promoting inflammation (118). Reduced YTHDF2 expression in SLE correlates with decreased C3, lower lymphocyte counts, and a higher neutrophil-to-lymphocyte ratio and promotes inflammation (119). Upregulated YTHDF3 and IGFBP2, alongside downregulated METTL14 and YTHDF2, disrupt MMP9 mRNA stability, enhancing expression, inflammation, and disease severity in SLE (120).

#### 4.1.2 m6A methylation in rheumatoid arthritis (RA)

RA is an immune-mediated disorder causing joint damage, bone erosion, and disability (63, 66). METTL3-driven m6A regulates the circINTS4/miR-146b-3p axis, where circINTS4 acts as a sponge to to suppress miR-146b-3p–driven FLS activation in RA (121). METTL3 in PBMCs and FLS, linked to elevated IL-6, MMP3, MMP9, TNF-α, CRP, ESR, and NF-κB-driven inflammation (69). METTL3-mediated m6A dysregulation in RA suppresses macrophage activation and regulates NF-κB and matrix synthesis in chondrocytes (100). In RA, METTL3 and YTHDF2 suppress PGC-1α, increasing ROS and cytokines in monocytes, while METTL3/YTHDC2 regulate PGC-1α and AMIGO2 to promote FLS proliferation and invasion (122). METTL3 stabilizes SLC7A11 via m6A-IGF2BP2 in RA, suppressing ferroptosis and driving FLS proliferation. Inhibiting METTL3 restores ferroptosis reduces synovial pathology (123). Beyond ferroptosis, cuproptosis is a novel m6A-regulated cell death in RA; Xinfeng Capsule suppresses the METTL3/miR-221/222-3p/ATP7A axis, reducing copper-induced chondrocyte death and inflammation (124). METTL3 upregulates RAC2 through m6A,activates AKT, and exacerbates RA cell proliferation, migration, oxidative damage, and inflammation (125). METTL14 deficiency reduces m6A in RA PBMCs/synovium, increasing disease activity; it destabilizes LASP1, inhibits TNF-α–SRC/AKT signaling, enhances cytokines, and suppresses TNFAIP3 via nuclear retention and mRNA decay (126). FTO and YTHDF2 correlate with IgG, C3, DAS28 in RA; reduced ALKBH5, FTO, and YTHDF2 in mononuclear cells indicate m6A disruption linked to ESR, CRP, RF (127). FTO demethylates lncRNA ENST00000619282, enhancing YTHDF1 expression, activating NF-κB, and promoting apoptosis resistance in RA synoviocytes (128). ALKBH5 knockdown inhibits FLS proliferation and invasion via JARID2 mRNA stabilization and CH25H modulation, reducing arthritis; SMOC2 suppresses MYO1C through ALKBH5/YTHDF2 m6A, affecting cytoskeleton (129, 130). ALKBH5 also downregulates NLRP3 via YTHDC2, further limiting RA progression (131). ALKBH5 modulates macrophage polarization by demethylating Itga4 mRNA in RA. Its piENOX2-mediated degradation elevates m6A, leading to PI3K-AKT activation and inflammation (132). WTAP promotes inflammation via the circ-0066715/miR-486-5p/ETS1 axis, while YTHDF2 loss stabilizes MAP2K4/MAP4K4, activating MAPK/NF-κB and increasing pro-inflammatory cytokines (71, 133). WTAP regulates macrophage polarization in RA by m6A-modifying exosomal circ-CBLB, reducing its levels and promoting M1 inflammatory activation (134). Mo et al. identified 37 m6A-associated SNPs linked to RA and 23 SNP–Gene–RA trios from gene expression data; further studies are needed to confirm their role in RA pathogenesis (135). m6A promotes proinflammatory RA M1 macrophages via cytokine secretion; 3-deazadenosine reduces inflammation by altering mitochondrial function and inhibiting macrophage migration and invasion (136, 137). In RA, dysregulated m6A-modified circRNAs and miRNAs control macrophage polarization and T cell responses by targeting NF-κB, JAK-STAT, and PI3K-AKT (138). Elevated IGFBP2 in RA FLS regulates growth hormone and natriuretic peptide receptor interactions. IGF2BP3 promotes proliferation via cyclin B1 and c-Myc, affects G2/M phase and macrophage polarization; IGF2BP3 stabilizes m6A-RASGRF1, activates mTORC1, promotes inflammation, inhibits autophagy, increases ROS and joint damage (139–141).

#### 4.1.3 m6A methylation in psoriasis (Ps)

Ps is a chronic immune-mediated condition influenced by genetic and environmental factors, affecting skin and sometimes joints; over 60 genetic susceptibility loci have been identified (142). Liu et al. analyzed Ps datasets (GSE30999, GSE13355) and identified ten m6A-related differentially expressed genes, with elevated YTHDC2 and reduced METTL3 and IGF2BP2 in Ps lesions, serving as biomarkers and therapeutic targets (143). m6A regulates lncRNA AGAP2-AS1, which sponges miR-424-5p to activate AKT3/AKT-mTOR signaling and promote proliferation; METTL3 stabilizes AGAP2-AS1 via YTHDF2, and its loss increases AGAP2-AS1, driving Ps progression (144). In contrast, METTL3 inhibition exacerbated Ps in an imiquimod-induced mouse model (145). METTL3-mediated m6A stabilizes SLC15A3 mRNA, activating TASL-IRF5-mediated M1 polarization in Ps, while ALKBH5 loss heightens inflammation (146). In Ps, m6A enhances keratinocyte proliferation and inflammation. Keratinocyte-derived CXCL1 and IL-6 intensify inflammation. IL-6 triggers METTL14-m6A methylation of TRIM27, stabilized by IGF2BP2, activating IL-6/STAT3 and amplifying keratinocyte pathology (147). In Ps lesions, lncRNA UCA1 activates HIF-1α and NF-κB via METTL14 to suppress inflammation, while METTL3-m6A-induced AGAP2-AS1 drives keratinocyte survival and proliferation through miR-424-5p/AKT/mTOR pathway (148).

In Ps patients, FTO, YTHDF1, and YTHDF2 mRNA levels rise and show positive correlation with C-reactive protein (149). ALKBH5 enhances psoriatic angiogenesis through AKT-mTOR in endothelial cells, exacerbating inflammation and lesion; ALKBH5 knockdown in CD4+ T cells worsens Ps-like disease, reversed by METTL3 restoration (150). Wang et al. used MeRIP-Seq to profile the m6A methylome in psoriatic skin, finding reduced m6A peaks and peak density in Ps-affected skin compared to healthy and unaffected skin. m6A sites with higher methylation were mainly in the coding region and 3’ UTR, while reduced methylation sites were enriched in the coding region, 3’ UTR, and 5’ UTR (151). YTHDF2 enhances Wnt signaling in Ps via m6A-DKK3 degradation, driving inflammation and proliferation (152). RBM15 upregulation in psoriatic skin stabilizes keratin 17 (K17), promoting keratinocyte proliferation and inflammation; RBM15 silencing reduces inflammation via K17 destabilization and IL-17A blockade; ALKBH5 knockdown in CD4+ T cells worsens Ps-like inflammation, reversed by METTL3. RBM15 upregulation in psoriatic skin stabilizes K17, promoting keratinocyte proliferation and inflammation; silencing RBM15 reduces inflammation via K17 destabilization and IL-17A inhibition; ALKBH5 knockdown in CD4+ T cells aggravates Ps-like inflammation, reversed by METTL3 expression (153). m6A regulates Ps via circRNA hsa-circ-0004287, elevated in inflammatory macrophages and PBMCs; it competes with IGF2BP3, destabilizes MALAT1, promotes S100A8/S100A9 degradation, and reduces p38 MAPK phosphorylation and inflammation (154). In CD4+T cells, m6A stabilizes IL-17A mRNA, enhancing IL-17 production and Ps progression (155). METTL3 m6A regulates γδ T cell balance via STAT1 degradation; its loss reduces γδ T17 psoriasis and IL-17 (156). Ps skin methylation analysis shows hypermethylated genes linked to immune response and cytokines, while hypomethylated genes associate with Wnt pathway and development (145).

#### 4.1.4 Comparative analysis of m6A modulation across systemic ADs (SLE, RA, Ps)

**Figure 4:**
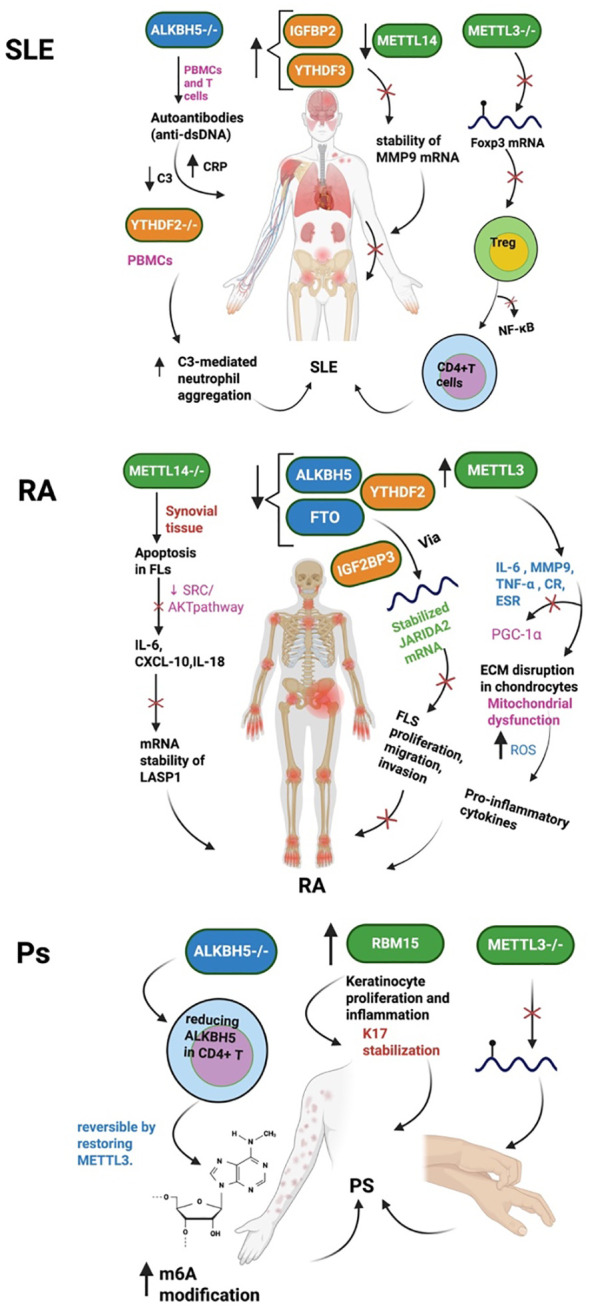
Differential expression of m6A RNA methylation-related enzymes in systemic ADS, including SLE, RA, and Ps, highlighting disease-specific patterns of writers, erasers, and readers.

Systemic ADs (SLE, RA, Ps) share m6A-related mechanisms despite clinical differences. In all three, METTL3 is upregulated, stabilizing pro-inflammatory cytokines (IFN-γ, CXCL2, IL-23, Foxp3) and enhancing Treg differentiation. Downregulation of demethylases (ALKBH5, FTO), leading to persistent inflammatory signaling. YTHDF2 consistently regulates cytokine levels by degrading transcripts (TNF-α, IFN-γ). Notably, dysregulation of IGF2BP proteins (IGF2BP2/3) stabilizes m6A-modified transcripts to drive inflammation and proliferation across these diseases (**Figure 4**). Despite shared m6A mechanisms, each disease exhibits distinct regulatory features. In SLE, ALKBH5 downregulation prolongs CD4+ T-cell activation and autoantibody production, while METTL3 promotes Treg function, mitochondrial homeostasis, and IRF4/PAX5-driven plasma cell-mediated renal damage; reduced YTHDF2 enhances NF-κB signalingIn RA, METTL3 activates NF-κB to amplify IL-6/TNF-α inflammation and suppresses ferroptosis via SLC7A11 stabilization, while METTL14 stabilizes TNFAIP3. ALKBH5 silencing restricts FLS invasion via JARID2 stabilization. In Ps, decreased METTL3/ALKBH5 impairs keratinocyte differentiation and boosts IL-6/STAT3 signaling via METTL14/TRIM27/IGF2BP2, while FTO downregulation enhances Th17 responses. YTHDF2 degrades DKK3 to activate Wnt inflammation, RBM15 stabilizes K17, and m6A stabilizes IL-17A mRNA in CD4+T cells. Unresolved mechanisms and distinct m6A targets highlight disease-specific therapeutic opportunities in SLE, RA, and Ps.

### 4.2 Organ-specific ADs: comparative analysis of m6A dynamics in T1DM, AITD, MS, and IBD

#### 4.2.1 m6A methylation in type 1 diabetes mellitus (T1DM)

In T1DM, genetic and environmental factors cause T cell-mediated β-cell destruction. Patients exhibit lower METTL3 and IGF2BP2, and higher YTHDC1, YTHDC2, hnRNPA2B1, hnRNPC, and ALKBH5 expression (157). Genetic variants in m6A regulators, such as the rs1862315 polymorphism in YTHDC2, have been implicated in increasing susceptibility to T1DM. METTL3 levels rise early but decline as T1DM progresses, promoting degradation of 2′−5′-oligoadenylate synthetase (OAS) mRNA via S-nitrosylation at cysteine residues C276 and C326 in its zinc finger domain. Sustained METTL3 expression protects β-cells by limiting OAS (158). In contrast, elevated METTL3 in diabetic nephropathy (DN) methylates TIMP2 mRNA, activating IGF2BP2-Notch signaling, triggering inflammation and apoptosis. In T1DM, loss of m6A in β cells destabilizes key genes (PDX1, MAFA, GCK, KCNJ11), disrupting insulin pathways and promoting dysfunction (159). In diabetes, METTL14-mediated m6A modification downregulates TPK1, IPMK, and PIK3R1 under thiamine-insulin therapy, enhancing glucose and lipid metabolism (160). The FTO rs9939609 variant modulates inflammation and lipid metabolism; variants increase childhood obesity and T2DM risk but not T1DM risk or onset, indicating a weight-independent role (161). FTO (rs9939609) and MC4R (rs17782313) polymorphisms modulate T1DM metabolism; MC4R regulates hypothalamic obesity pathways and FTO affects adipocyte differentiation and insulin sensitivity (162). FTO-mediated m6A demethylation destabilizes antioxidant transcripts in β-cells, worsening oxidative stress and autophagy in T1DM (163). FTO demethylates NLRP3 mRNA, reducing pyroptosis and kidney injury in diabetic kidney disease (164). FTO regulates diabetic retinopathy by m6A-stabilizing MAFG-AS1, reducing endothelial dysfunction and inflammation (165). ALKBH5 reduces diabetic retinopathy by destabilizing ACSL4 mRNA via YTHDF1, suppressing ferroptosis (166). In STZ-T1DM mice, decreased hippocampal YTHDF1/3 and WTAP correlate with cognitive deficits; AAV-YTHDF1 overexpression improves memory and learning (157). YTHDF1 supports β-cell survival and reduces T1DM cognitive decline by promoting m6A-dependent autophagy via ATG2A/ATG5/ATG14, enhanced by HIF-1α-rich MSC vesicles (167). Genetic variants in YTHDC2 (rs1862315) and PRRC2A, particularly in non-coding regions, may modulate immune m6A methylation and T1DM risk (160). In DN, METTL3 downregulates circ-0000953, impairing autophagy and increasing podocyte injury via the miR-665-3p-Atg4b axis (168). Elevated RBM15 activates the AGE-RAGE pathway, promoting inflammation and pyroptosis (169). WTAP increases m6A on NLRP3 mRNA, triggering inflammasome activation and pyroptosis (170).

#### 4.2.2 m6A methylation in autoimmune thyroid disease (AITD)

AITD is recognized as one of the most prevalent autoimmune disorders, characterized by significant heritability (171). Research has revealed various genetic loci associated with AITD autoimmunity (5). High-throughput sequencing revealed associations between METTL3 gene polymorphisms and AITD susceptibility risk at multiple loci (77, 172). A case-control study (979 AITD, 732 controls) found ALKBH5 polymorphism strongly associated with AITD; variants alter m6A demethylation, affecting T cell activation and autoimmunity (173). Song et al. identified ALKBH5 variants (rs9913266, rs12936694) linked to AITD, suggesting genetic susceptibility (174). Graves’ disease (GD) and Hashimoto’s thyroiditis (HT show distinct m6A profiles; in GD, METTL3 suppression increases SOCS expression in CD4+ T cells, indicating immune regulation (175). In HT, m6A modification of ATF4 by hnRNPC induces ER stress in thyroid cells, promoting apoptosis and necrosis (98, 176). m6A-dependent epitranscriptomic control of hub genes such as ITGAM, CD86, and IL10RA influences autoimmune responses in GD (177).

#### 4.2.3 m6A methylation in multiple sclerosis (MS)

MS is an autoimmune disease marked by chronic CNS inflammation, demyelination, and nerve damage (64). Relapsing-remitting MS (RRMS) and progressive forms of multiple sclerosis (PMS), such as primary progressive MS and secondary MS, are recognized classifications (178). The potential susceptibility loci for MS have been identified in over 200 genomic regions (179). m6A modification in cerebrospinal fluid is being explored as a potential PMS diagnostic marker. MS patients show elevated expression of m6A regulators compared to non-MS controls across 13 centers. RRMS samples exhibit higher m6A levels than PMS (64). Upregulated METTL3 increases m6A, stabilizing CXCL2 and IFN-γ mRNAs to enhance cytokines, T-cell activation, and CNS infiltration; METTL3/IGF2BP2 stabilize IκBα mRNA via m6A, promoting M2 microglia and reducing CNS inflammation (180). In addition to T cell effects, METTL3 in B cells plays a protective role in MS; B cell-specific METTL3 knockout exacerbates EAE, while overexpression promotes regulatory B cell differentiation and suppresses pro-inflammatory cytokines (88). METTL3-driven m6A of BATF promotes microglial activation and neuroinflammation in MS (181). METTL3 promotes CNS microglia (M1/M2) inflammation via TRAF6-NF-κB; its loss in EAE stabilizes SOCS3, reduces CCR5/IL-17A and Th17 activity, limiting CNS damage (182, 183). METTL14 loss oligodendrocytes causes mRNA hypomethylation of transcription factors (Hey1, Klf19, Zeb2), histone modifiers (HDAC3, KDM2B, PRDM2), and growth factors (IGF-1, FGF, BMP), impairing differentiation, maturation, myelination, RNA splicing, and reducing neurofascin variants, worsening Ranvier node formation and MS pathology (184). In EAE, ALKBH5 deletion in T cells lowers m6A on CXCL2 and IFN-γ mRNAs, increasing their stability, protein expression, CD4+ T-cell responses, and CNS inflammation (33).

A significant association was found between the FTO rs9939609 A-allele and elevated homocysteine levels in individuals with MS. Another study reported a correlation between rs9939609 and heightened disability in individuals with MS (185). Nevertheless, neither study directly linked FTO variants with MS risk. In contrast, a notable link was observed between the FTO variant rs1558902 and MS risk in Hispanic populations (186). Utilizing data from GWAS and QTL studies, two key genetic variants were identified: rs923829 within METTL21B and rs2288481 within DKKL1, both significantly linked to MS. Strong links were found between MS and non-MHC genes (METTL21B, METTL1, TSFM), with DDR1 SNPs affecting MICB and granzyme A levels, suggesting m6A proteins play a role in MS (187). Comparative analysis of 61 cerebrospinal fluid samples from MS patients alongside 31 controls showed elevated expression of 13 key m6A regulators in MS, suggesting their potential as biomarkers and their role in ADs (188). Future studies should strive to elucidate the way m6A RNA methylation modulates MS processes and its intricate mechanisms.

#### 4.2.4 m6A methylation in inflammatory bowel disease (IBD)

IBD, a chronic inflammatory disease that recurs periodically, includes both Crohn’s disease (CD) and ulcerative colitis (UC), posing a significant global healthcare burden with increasing prevalence worldwide. Comprehensive genetic analyses have identified numerous susceptibility genes linked to IBD (65). METTL3-mediated m6A stabilizes UAF1 mRNA, boosting its expression and NLRP3 inflammasome inflammation in colitis. Targeting UAF1 reduces severity and restores the intestinal barrier (189). METTL3 is upregulated in IBD patients and DSS mice; knockdown alleviates DSS-induced IBD via NF-κB suppression (190). Tong et al. found that Foxp3-mediated METTL3 deletion in Tregs causes severe autoimmune responses; m6A–XPO1–NF-κB pathway activation in CD highlights m6A regulation in IBD and METTL3’s role in immune balance and gut homeostasis (72).

Murine experiments using CD4-Cre transgenic mice with METTL14 floxed alleles (CD4-Cre+/Tg METTL14FL/FL) conditional knockout models show METTL14 deficiency impairs iTreg differentiation via reduced RORγt, leading to spontaneous colitis and disruption of Th1/Th17/Treg balance, highlighting m6A’s role in Treg stability (191). METTL14 deficiency in intestinal epithelial cells increases NFKBIA, promoting TNF-induced apoptosis and barrier damage; it also stabilizes GPX4 mRNA, preventing ferroptosis and oxidative injury in UC (192). Zhou et al. showed that after ATC induction, ALKBH5-deficient CD4+T cells (ALKBH5flox/floxCd4Cre) had reduced colonic infiltration compared with WT cells, indicating ALKBH5 deletion prevents T-cell accumulation in colon tissue; m6A modification contributes to IBD risk via gene expression changes (33). In UC, FTO downregulation reduces CerS6 stability, causing sphingosine-1-phosphate accumulation, M1 macrophage polarization, and Th17 differentiation, exacerbating colitis; YTHDC1 loss boosts M1 and Th1/Th17 responses via RhoH/NF-κB dysregulation (193). FTO SUMOylation alters m6A demethylation in IBD, redirecting BMSC differentiation from osteogenesis to adipogenesis (194).

YTHDC1 deletion in macrophages and reduced IGF2BP2 expression collectively enhance M1 polarization and proinflammatory Th1/Th17 cell activity in the gut, exacerbating intestinal inflammation (195). YTHDC1 maintains barrier integrity via NME1, while METTL3-driven m6A on circPRKAR1B triggers autophagy and NLRP3 pyroptosis in CD progression (195). Chen et al. found decreased IGF2BP1/IGF2BP2 in CD and lower IGF2BP2 in UC; m6A hub genes (NUP37, SNRPG, H2AFZ) were increased in macrophages and naive B cells, suggesting effects on immune infiltration and treatment response. The rs11498 SNP affects YTHDC1 binding to m6A-marked LOC339803, shifting its chromatin position and activating NF-κB–driven pro-inflammatory cytokines (196). In line with these results, Wang et al. observed exacerbated development of DSS-induced colitis in IGF2BP2 knockout mice, highlighting the crucial function of IGF2BP2 in IBD (197). IGF2BP2 stabilizes LGI4 mRNA via m6A in intestinal epithelial cells, inhibiting MEK1/2–ERK1/2 signaling and cytokines; its loss worsens UC, reversible by LGI4 restoration. IGF2BP2 also stabilizes CBR1 mRNA, suppressing PI3K/Akt/NF-κB activation; reduced IGF2BP2 increases disease severity (198). hnRNPC is upregulated in macrophages during DSS colitis, modulating inflammation by alternative splicing and stabilizing Itgb7 mRNA, influencing inflammation and cell migration (199).

#### 4.2.5 Comparative analysis of m6A modulation across organ-specific ADs (T1DM, AITD, MS, IBD)

**Figure 5:**
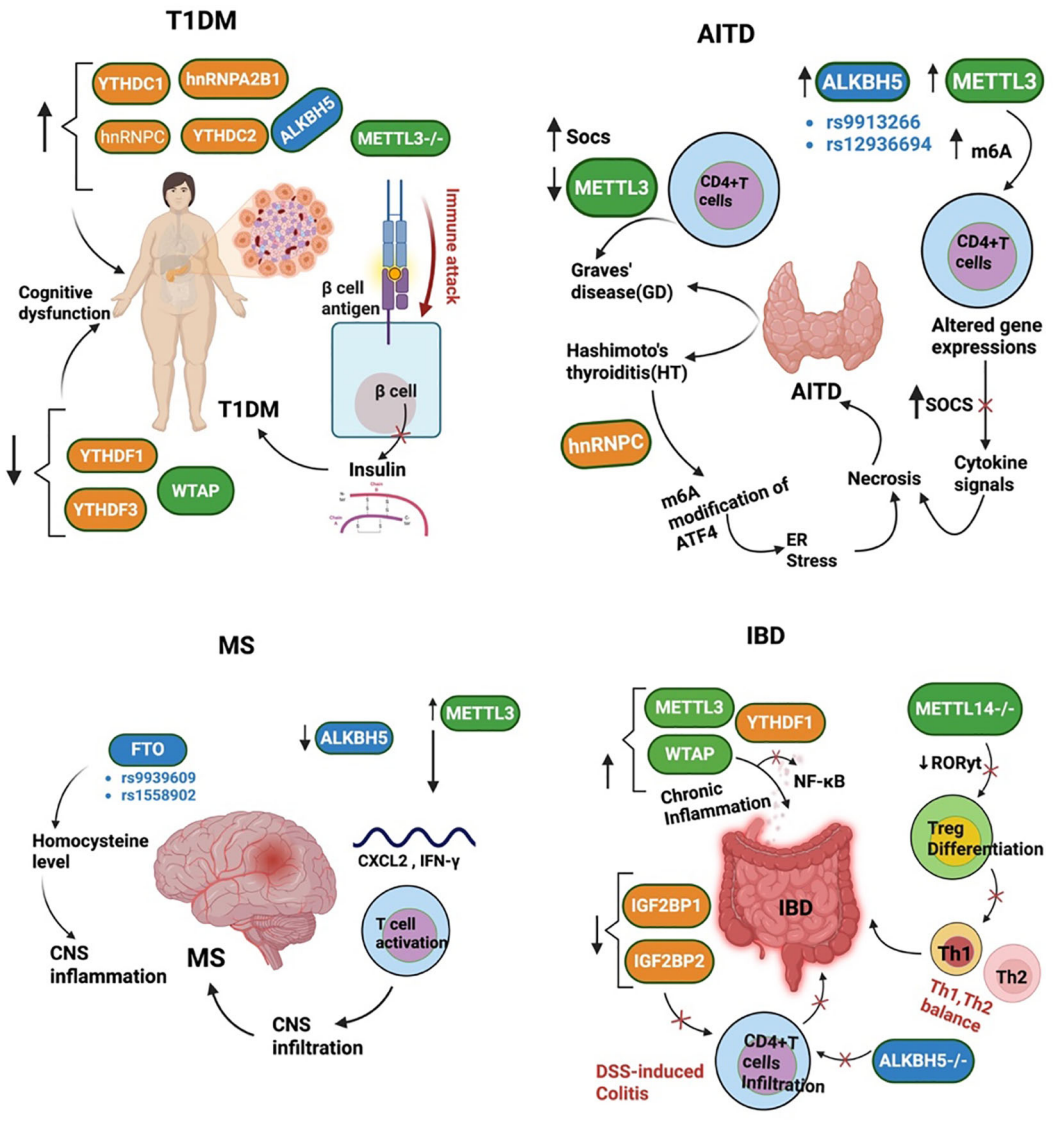
Expression landscape of m6A regulators in organ-specific ADS, including T1DM, AITDs, and IBD, emphasizing distinct m6A-associated alterations across tissue-targeted autoimmune responses.

m6A regulators have shared and disease-specific roles in organ-specific ADs (T1DM, AITD, MS, IBD), modulating inflammation via NF-κB, IL-6, and STAT pathways (**Figure 5**). In T1DM, m6A promotes β-cell apoptosis by destabilizing insulin mRNA and sustaining the IFN-γ response through decreased ALKBH5, while METTL3 contributes to CXCL10 stabilization. In AITD, METTL3 and METTL14 regulate inflammatory signaling that causes thyroid damage, with reduced FTO sustaining thyroid inflammation; however, their roles in GD versus HT, TSH receptor pathways, and autoantibody production remain unclear. In MS, increased METTL3 drives persistent IFN-γ production via microglia, enhances T-cell activation, and disrupts the blood-brain barrier, but its functions in oligodendrocytes, relapse/remission phases, and cytokine modulation such as CXCL2 and IFN-γ require further investigation. In IBD, m6A expression fluctuates during flare-ups, with decreased METTL14 impairing epithelial barrier integrity and shifting macrophages from M2 to M1, while increased METTL3 promotes immune cell infiltration and its effects on gut microbiota, cytokines, and epithelial cells remain unclear. Although these diseases share chronic inflammation, APC dysregulation, and cytokine signaling, they differ in target tissues, immune pathways, and m6A regulator dynamics. METTL3, ALKBH5, and FTO have distinct roles, and disease-specific targets such as INS/OAS in T1DM, NLRP3/ATF4 in AITD, CXCL2/SOCS3 in MS, and HIF-1α/CerS6 in IBD highlight divergent m6A-driven outcomes.

Critical gaps remain, including the lack of β-cell-specific m6A therapies, limited data from human thyroid and MS tissues, minimal understanding of m6A in BBB permeability and T-cell migration, and unexplored gut microbiota-m6A interactions in IBD. Current limitations include a lack of longitudinal and single-cell profiling, insufficient studies on non-coding RNAs, and high patient variability. Future directions involve ALKBH5-based therapies to protect β-cells in T1DM, organoid models and m6A inhibitors in AITD, m6A-based biomarkers and METTL3/YTHDF2 research in MS subtypes, and targeting m6A-microbiome crosstalk and epithelial repair strategies in IBD.

### 4.3 m6A in rare studied immune diseases

#### 4.3.1 m6A methylation in myasthenia gravis (MG)

MG shows increased RBM15 and YTHDF1 expression affecting histone modification and Wnt signaling; RBM15 is strongly linked to immune profiles (200). Downregulated m6A-modified circRNAs (hsa-circ-0084735, hsa-circ-0025731) disrupt immune signaling, suggesting biomarker potential (201).

#### 4.3.2 m6A methylation in Celiac’ disease

In celiac disease, the m6A–XPO1–NF-κB pathway is activated; gliadin increases XPO1 translation via m6A/YTHDF1 in intestinal cells (202). Allele-specific variations in XPO1 in celiac patients enhance gluten-dependent m6A signaling and inflammation, supporting therapeutic targeting (72). ncRNAs (miRNAs, lncRNAs, circRNAs) regulate immune pathways in celiac disease, serving as biomarkers and inflammation modulators (203). METTL3 inhibition reduces m6A on IRF7 transcripts, impairing YTHDC2 antiviral signaling and inflammatory gene expression; similar effects are seen with simvastatin (204).

#### 4.3.3 m6A methylation in uveitis pathogenesis

Uveitis is an inflammatory eye disease with limited study on m6A. In autoimmune uveitis, retinal pigment epithelial cells show reduced FTO. FTO knockout in ARPE-19 cells increased m6A on ATF4 mRNA, lowering ATF4 protein and promoting cell proliferation and secretion of IL-6, IL-8, and MCP-1 (205). FTO-mediated m6A demethylation stabilizes miR-31-5p, suppressing M1 macrophage polarization via P2RX7 targeting and p38 MAPK inhibition in autoimmune dry eye (74). m6A regulators (FTO, YTHDF2, CBLL1, METTL14) control immune gene regulation and clinical heterogeneity in Behçet’s uveitis (206).

#### 4.3.4 m6A methylation in ankylosing spondylitis (AS)

AS is a chronic inflammatory disease affecting spine and joints, causing ligament calcification and synovial overgrowth (207). TNF-α promotes AS-MSC migration and pathogenesis by increasing ELMO1 expression, while METTL14 regulates MSC migration via m6A modification of ELMO1 3’UTR (208). METTL14-mediated m6A stabilizes FOXO3a mRNA, enhancing autophagy and reducing AS inflammation (209). YTHDF3 stabilizes IL32 mRNA and promotes osteogenic differentiation in AS bone mesenchymal stem cells (210). ALKBH5 downregulation in AS-PBMCs increases m6A and IGF2BP3-driven NRG1 mRNA stability, suppressing autophagy and disease (211). Elevated FTO in AS MSCs reduces NORAD m6A methylation, stabilizing NORAD and blocking osteoclast formation via miR-4284 (212).

#### 4.3.5 m6A methylation in scleroderma

MeRIP-qPCR in scleroderma mice revealed higher methylation of H-ras, LAMA3, TNC, and lower methylation of CCL3, CCL9, SAA1, IL1B, indicating m6A’s involvement in fibrosis and inflammation pathways (213). In bleomycin-induced scleroderma mice, FTO overexpression reduced m6A and TNC mRNA levels, lessening skin fibrosis and highlighting the FTO/TNC pathway as a therapeutic target (214, 215).

#### 4.3.6 m6A methylation in autoimmune hepatitis (AIH)

In AIH, YTHDF2 upregulation correlates with increased MDSCs. YTHDF2-deficient mice showed increased liver MDSCs, protecting against damage and promoting liver enlargement and migration (216).

## 5 Therapeutic potential of m6A targeting in autoimmunity

### 5.1 Clinical unmet needs motivating m6A-targeted therapeutics

Despite its therapeutic potential, key challenges must be addressed before clinical application. m6A-related enzymes exhibit paradoxical roles in ADs, either promoting or inhibiting disease progression. Furthermore, m6A-mediated effects differ across animal models, as METTL3 or METTL14 depletion in T cells may produce opposing outcomes through distinct signalling pathways (80, 191). Similar findings have been reported in RA, where METTL14 knockdown in mice synovium produced contrasting effects (217). Variable expression of m6A-related enzymes in PBMCs further indicates that m6A modification may exert distinct effects across different stages and levels of disease activity (218). Furthermore, the immune microenvironment may influence m6A modification. For example, hypoxia upregulates ALKBH5 in FLSs. Thus, the functions of m6A enzymes may vary with the immune microenvironment, disease status and underlying molecular mechanisms, reflecting the complexity of epigenetic regulation. Although progress has been made in exploring the diagnostic value of m6A methylation in early-stage diseases, further studies are needed to validate these findings. A deeper understanding of its effects on immune responses is required before targeted interventions. The methodologies discussed may introduce biases, as antibody-based enrichment and high-throughput sequencing may not fully capture transcriptomic complexity, leading to false-positive or false-negative findings. Moreover, the heterogeneous and highly plastic nature of macrophage polarization, which extends beyond the classical M1/M2 paradigm, further complicates the interpretation of m6A-mediated immune regulation. Animal studies show the therapeutic efficacy of m6A inhibitors, but their use as a therapeutic strategy remains in the experimental phase with limited clinical trials. Most studies combining m6A inhibitors with immunotherapy are cancer-focused, with little data in ADs on efficacy and toxicity. Moreover, m6A targeting may induce excessive immune activation, requiring further validation. Further mechanistic insights will advance precision medicine for ADs.

### 5.2 m6A targeting and immune checkpoint therapy

Immune checkpoints are inhibitory regulators that act as “brakes” to maintain immune homeostasis and self-tolerance by modulating the intensity and duration of immune responses via ligand binding. Key checkpoints include PD-1, PD-L1, CTLA-4, and TIM-3. PD-1/PD-L1 suppress immune responses by recruiting phosphatases that reduce T-cell activation, while CTLA-4 competes with CD28 for B7 ligands to inhibit T-cell activation. m6A modification regulates immune checkpoint expression, especially in tumor immune escape and immune tolerance. METTL3 enhances PD-L1 mRNA stability and transcription via IGF2BP3-dependent m6A recognition, suppressing CD8^+^T-cell-mediated antitumour immunity (219). It upregulates PD-L1 expression through the lncRNA MALAT1 pathway in pancreatic cancer, supporting immune checkpoint-mediated tumour progression (220). Additionally, METTL3-mediated m6A modification of circIGF2BP3 upregulates PKP3 and stabilizes OTUB1 mRNA, increasing PD-L1 abundance through reduced ubiquitination and thereby facilitating tumour immune evasion (221). METTL14-mediated m6A modification of Siah2 promotes its YTHDF2-dependent degradation, inhibiting PD-L1 ubiquitination and increasing PD-L1 expression, thereby contributing to immune escape (222). It also enhances PD-L1 expression through m6A modification of MIR155HG and regulation of the miR-223/STAT1 axis, facilitating immune evasion in hepatocellular carcinoma (223). FTO-mediated m6A demethylation induces PD-1 expression through the autophagy/NF-κB/FTO axis, suppressing anti-PD-1 responses (224). Moreover, FTO depletion decreases PD-L1 expression in colon cancer, indicating its positive regulation of PD-L1 (225). ALKBH5-mediated m6A demethylation inhibits PD-L1 expression on macrophages, regulating T-cell infiltration and cytotoxicity (173, 226). YTHDF1-mediated m6A regulation impairs dendritic cell antigen presentation and CD8+ T-cell infiltration, while YTHDF1 depletion enhances antitumour immunity and anti-PD-L1 efficacy (52). Abatacept, a CTLA-4-targeting drug, modulates T cell co-stimulatory signals to reduce immune inflammation and is approved for adult RA (227).

### 5.3 m6A as a diagnostic and therapeutic target

ADs complexity requires targeted therapies; epigenetic biomarkers are crucial for disease tracking and therapy development (**Table 3**). METTL3 inhibitors (STM2457/knockout) and have been reported to suppress inflammatory mediators, ECM degradation, apoptosis, autophagy, ferroptosis, and pyroptosis via SLC7A11 destabilization, leading to reduced synovial hyperplasia and bone damag offering a novel therapeutic approach (228–231). METTL3 targeting disrupts m6A-dependent TGF-β-induced dermal fibrosis, offering systemic sclerosis treatment (232). Moreover, WKYMVM, a METTL14 inhibitor, suppresses Th1 and Th17 differentiation, reduces paw swelling, and lowers inflammatory cytokine production (233). Intra-articular lentiviral shMETTL3 reduced arthritis severity and inhibited aggressive synovial fibroblast (SF) behavior in CIA mice (123). FB23-2, an FTO inhibitor, reduced arthritis scores, paw thickness, synovial inflammation, cartilage damage, and bone destruction, supporting its therapeutic potential in RA. Similarly, intra-articular FTO shRNA injection significantly mitigated arthritis severity (234). In patients with SLE and active cutaneous lupus erythematosus (CLE), the FTO inhibitor mupirocin reduced skin inflammation (235). FTO reduces m6A on TNC mRNA, lowering fibrosis and suggesting scleroderma therapy potential. Natural inhibitors (Rhein, meclofenamic acid, R-2-hydroxyglutarate, CS1/CS2) block m6A demethylation and enhance cytotoxic lymphocyte function (236). FTO polymorphisms associated with adverse purine therapy reactions in IBD identify m6A regulators as potential autoimmune targets (237). CS2 (FTO inhibitor) combined with anti-PD-1 enhances CD45^+^F4/80^+^ macrophage, especially antitumoral M1, infiltration, suppressing tumor growth in a spontaneous HCC model (238). Inhibition of ALKBH5 by Xinfeng Capsule restores m6A methylation of LINC00968, thereby suppressing neutrophil hyperactivation, NET formation, and oxidative stress–mediated inflammation in RA (239). Curcumin, an ALKBH5 inhibitor, increased antioxidant enzyme activity, reduced pro-oxidant levels, and alleviated RA disease activity (240). ALK-04, an ALKBH5 inhibitor, reduces tumor growth and enhances immunotherapy by modulating immune infiltration. ALKBH5 variants are linked to AITD; deletion reduces T-cell infiltration in IBD, inhibition alleviates EAE, while knockdown worsens psoriasis, suggesting therapeutic potential in ADs (241). m6A promotes proinflammatory RA M1 macrophages via cytokine secretion; 3-deazadenosine reduces inflammation by altering mitochondrial function and preventing migration (70).

**Table 3:** Potential m6A regulators as therapeutic targets in ADs.

m6A regulator/inhibitor	Target cells	ADs involved	Disease stage	Main immunological effect	Therapeutic potential	Strength of preclinical evidence	References
METTL3 (STM2457, METTL3 knockout)	Th17 cells, DCs	MS	Early/Progression	Promotes Th17 differentiation via STAT3; drives EAE.	Strong inhibition reduces Th17-mediated inflammation in MS.	*In vivo*	(123, 228–233)
	T cells	SLE	Early	Enhances T cell activation & survival.	Potential combo with IL-17/IL-23 blockade.	*In vitro*	
	DCs	RA	Progression	Promotes antigen presentation and inflammation.	modulates T cell activation in SLE; prevents RA tissue damage; antifibrotic effect in systemic sclerosis.	*In vivo* + *In vitro*	
METTL14 (WKYMVM, siRNA)	CD4+T cells, intestinal DCs	IBD	Progression	Enhances PD-1/PD-L1 signaling by increasing m6A modification of immune checkpoint mRNAs in intestinal T cells, leading to T cell exhaustion and decreased inflammation.	Enhances PD-1/PD-L1 checkpoint sensitivity in IBD and AS.	*In vivo*	(233)
	CD4+T cells, spinal APCs	AS	Progression	Upregulates PD-L1 expression on spinal antigen-presenting cells, immune checkpoint	Potential for PD-1-resistant autoimmune conditions.	*In vitro*	
FTO (FB23-2, Rhein, Meclofenamic Acid, R-2HG, CS1/CS2, DAC51, Mupirocin, FTO-shRNA)	Osteoblasts,	RA,	Progression	Demethylates TNC mRNA; miR-223p pathway, suppressing bone formation and contributing to joint damage.	Regulates osteoblast differentiation, reducing bone damage in RA.	*In vivo* + *In vitro*	(234–238)
	Fibroblasts	Scleroderma	Progression.	Promotes fibrosis by demethylating fibrosis-associated genes (e.g., TNC).	Anti-fibrotic potential in scleroderma.	*In vitro*	
	T/B cells	IBD	Late (Resistance)	SNPs linked to poor response to thiopurine therapy.	May overcome thiopurine resistance in IBD.	Genetic association	
ALKBH5 (ALK-04), (Curcumin)	T cells, APCs	IBD	Progression	Inhibits gut inflammation by reducing T cell infiltration; modulates APC activity.	Alleviates gut inflammation by reducing T cell infiltration in IBD.	*In vivo*	(239–241)
	Skin APCs	Psoriasis	Flare	ALKBH5 inhibition decreases inflammatory gene expression; deletion worsens disease.	Modulates flare in psoriasis.	*In vivo*	
	CNS APCs	EAE	Progression	Suppresses Th17-mediated neuroinflammation; promotes immune tolerance.	Improves neuroinflammation in EAE/MS.	*In vivo*	
YTHDF1 (LNP-siYTHDF)	B cells	SLE	Chronic	Stabilizes IgG1 mRNA in antibody-secreting plasma cells.	Stabilizes IgG mRNA to maintain antibody levels in SLE.	*In vivo*	(242–245)
	Neurons	T1DM	Chronic/Cognitive	Promotes synaptic gene translation; improves hippocampal plasticity.	Potential target for cognitive dysfunction in T1DM.	*In vivo*	
YTHDF2 DC-Y13-27, DF-A7)	MDSCs	AIH, Liver Inflammation	Flare	Deficiency enhances MDSC-mediated protection, reduces liver inflammation in AIH.	Enhances MDSC function to reduce liver inflammation in AIH.	*In vivo*	
YTHDC1 Ebselen, experimental inhibitors)	B cells, plasma cells	SLE	Early & Chronic.	Regulates mRNA splicing and export in activated B cells and plasma cells, influencing autoantibody (IgG, IgA) production and B cell survival.	Regulates immune cell mRNA splicing in SLE and RA.	*In vitro*	(246)
	T cells, synovial macrophages	RA	Chronic Synovial Phase	Modulates mRNA splicing of proinflammatory cytokines (e.g., IL-6, TNF-α) in T cells and macrophages, contributing to synovial inflammation	May synergize with B cell–targeted biologics.	*In vitro*	
IGF2BP3(Triptolide, Berberine, TwHF)	RA-FLS, T cells	RA	Chronic Synovial Phase	Promotes synovial proliferation, modulates G2/M transition. TwHF (triptolide) offers novel therapy for RA.	Suppresses synovial proliferation in RA; novel anti-inflammatory effect; potential TwHF-based therapy.	*In vivo* + *In vitro*	(141, 247, 248)

YTHDF1 regulates IgG heavy-chain mRNA stability and IgG1 levels in SLE, with reduced expression after immunosuppression, while disrupting YTHDF1-m6A interaction alleviates lupus symptoms in mice in B cells (242). In T1DM, YTHDF1 promotes m6A-mediated synaptic gene translation and cognition; dysregulation impairs hippocampal activity, suggesting a target for diabetic cognitive issues (243). Silencing YTHDF1 via lipid nanoparticles (LNP-SIYTHDF1) combined with anti-PD-1 enhanced antitumor immunity by reducing immunosuppressive MDSCs and increasing IFN-γ^+^ and Granzyme B^+^ CD8^+^ T cells in MC38, CT26, and NASH-HCC models (244). YTHDF1 deficiency combined with anti-CTLA-4 or anti-PD-L1 boosted T cell immunity and improved survival (245). YTHDF2 deficiency could enhance MDSC-mediated protection and reduce liver inflammation in AIH (216). Inhibiting YTHDF2 with DC-Y13-27, which blocks its binding to m6A transcripts, or DF-A7, promoting its degradation, significantly improved ICI efficacy. DC-Y13–27 plus anti-PD-L1 and radiotherapy induced the strongest immune activation. In the liver, YTHDF2 depletion increased CX3CL1-driven CD8+T cell infiltration, improving combined oxaliplatin and anti-PD-1 therapy (249). Loss of YTHDF2 enhances Th9 differentiation by stabilizing Gata3 and Smad3 mRNAs under IL-4 and TGF-β signaling, increasing IL-9 and IL-21 production, CD8+ T and NK cell infiltration, and antitumor efficacy. In CAR-Th9 cells, YTHDF2 depletion sustains immune activation and cytotoxicity, identifying it as a negative epitranscriptomic checkpoint in T cell immunity (3). YTHDC1 inhibitor ebselen also improved RA symptoms (246). IGF2BP3 knockout and its inhibitor berberine alleviated RA manifestations (141), IGF2BP3 inhibition by berberine also reduced inflammation in monocytes from SLE patients (247). In RA, IGF2BP3 stabilizes transcripts promoting RA-FLS proliferation via the G2/M transition (248). Tripterygium wilfordii Hook F (TwHF), containing triptolide (TP), binds IGF2BP3, reducing its mRNA expression in PBMCs and RA fibroblast-like synoviocytes (MH7A), limiting synovial inflammation and offering a novel RA therapy (130). Berberine enhanced islet β-cell proliferation in T1D mice and reduced inflammation in EAU rats, while β-cell–specific METTL3 overexpression partially reversed T1D abnormalities (250, 251).

m6A methylation levels differ between IBD, MS, psoriasis, AITD, SLE, RA and Ps and healthy controls. This differential expression suggests the potential of peripheral blood m6A levels for early diagnosis. For instance, m6A RNA methylation distinguishes PMS from RRMS early, serving as a CSF diagnostic marker (64). The hypermethylated circRNA hsa_circ_0007259 activates the STAT3 pathway via hsa_miR-21-5p, contributes to RA pathophysiology and may serve as a diagnostic biomarker (252). Furthermore, GEO analysis identified five differentially expressed m6A regulators in RA. An m6A system distinguished between RA C1 and C2 subtypes (associated with higher inflammatory responses), highlighting its potential as a biomarker of inflammatory activity (253).

### 5.4 Challenges in m6A modulation

Despite remarkable advances in m6A regulation in autoimmunity, mechanistic and clinically understanding remains incomplete. Nevertheless, the dual roles of RNA-modifying enzymes complicate therapeutic targeting. The same factor can act as either oncogenic or tumour-suppressive depending on cellular context. For example, METTL3 exhibits opposing pro- and anti-inflammatory roles across immune cells depending on cell type, disease stage, and inflammatory microenvironment (75, 100). Similarly, YTHDF1 enhances antigen cross-presentation in some settings but suppresses it in others (52). These discrepancies reflect true biological complexity and require systematic study using cell-type-specific delivery systems such as targeted nanoparticles (254), or virus-like particles, and temporal control tools including drug-inducible degrons or switchable RNA editors, conditional knockout models and longitudinal patient sampling. Human autoimmune tissues (RA synovium, MS lesions, IBD gut) contain macrophages expressing both M1 and M2 markers simultaneously. Future studies must move beyond binary classification and adopt continuum-based or single-cell approaches to capture true macrophage heterogeneity. Most m6A inhibitor studies are based on cancer models, with limited validation in autoimmune diseases. Whether METTL3 inhibition (STM2457) or FTO inhibitors (Rhein, DAC51) can modulate autoimmunity without excessive immune activation remains unclear. Long-term safety and off-target effects on non-immune tissues require further evaluation. Differential m6A regulator expression has been reported in PBMCs of SLE, RA, and MS patients, but no validated m6A-based biomarker exists for early diagnosis, disease activity monitoring, or treatment response prediction. Longitudinal studies linking m6A signatures with clinical outcomes are urgently needed. m6A inhibitors synergize with JAK inhibitors, TNF blockers, or CTLA-4 agonists (abatacept) to restore immune tolerance is unknown. Combinatorial approaches may enable precision medicine but require systematic preclinical validation.

## 6 Conclusion

m6A is a key epitranscriptomic regulator integrating transcriptional, metabolic, and post-transcriptional programs to control immune activation, differentiation, and functional plasticity in ADs. Through coordinated actions of writers, erasers, and readers, m6A reshapes transcript stability and translation, thereby linking environmental and inflammatory cues to precise immune gene-expression programs that govern immune homeostasis and tolerance. The primary objective of this review is to synthesize current knowledge on m6A RNA methylation in immune cell regulation and ADs. We compare m6A mechanisms across innate and adaptive immune cells, analyze systemic versus organ-specific ADs, critically evaluate therapeutic strategies, and highlight unresolved controversies and knowledge gaps to provide a unified framework for future research and clinical translation. By integrating evidence from diverse autoimmune diseases, this review aims to establish a unified conceptual framework that bridges molecular epitranscriptomics with immune dysregulation, thereby guiding future diagnostic and therapeutic strategies.

## 7 Future directions in m6A-targeted therapeutics

Most current studies analyze individual RNA modifications in limited subsets, lacking developmental and microenvironmental context. This fragmented approach obscures stage-specific effects, as the same modification may promote proliferation during activation but restrain memory maintenance later. The future of m6A biology lies in bridging mechanistic insight with clinical translation to enable precise therapeutic targeting in immune diseases. To overcome these challenges, gene-level targeting should be prioritized by focusing on aberrantly expressed m6A regulators (YTHDF2 in pSS) (255), or downstream cell-restricted effectors. This strategy preserves physiological m6A function while enhancing therapeutic specificity. Single-cell and direct RNA sequencing technologies should be systematically integrated, while low-input, high-sensitivity detection platforms need adaptation for clinical workflows. Nanopore direct RNA sequencing now enables single-molecule and single-nucleotide resolution of multiple RNA modifications, providing a unified, pretreatment-free framework for quantitative profiling and facilitating efficient clinical translation (256). Integration of machine-learning–driven basecalling algorithms with harmonized cloud-based analysis pipelines is expected to enable reproducible quantification and support regulatory standardization in future epitranscriptomic studies (257). For immunological applications, clinical standardization of single-cell epitranscriptomics will require three key components: (1) reference cell lines and synthetic spike-in controls to calibrate sensitivity and ensure inter-laboratory reproducibility (258), (2) automated low-input library preparation workflows integrating direct RNA sequencing with optimized barcoding and multiplexing for limited patient samples (259), and (3) standardized computational pipelines and annotation frameworks for basecalling, signal normalization, and modification detection (260). Together, these developments will enable the construction of a spatiotemporal epitranscriptomic atlas, facilitating causal analysis of stage-specific m6A-modification events through inducible perturbation models in immune-mediated diseases.
